# Finerenone in Hispanic Patients With CKD and Type 2 Diabetes: A Post Hoc FIDELITY Analysis

**DOI:** 10.1016/j.xkme.2023.100704

**Published:** 2023-08-01

**Authors:** Sylvia E. Rosas, Luis M. Ruilope, Stefan D. Anker, Bertram Pitt, Peter Rossing, Andres Angelo Cadena Bonfanti, Ricardo Correa-Rotter, Fernando González, Carlos Francisco Jaramillo Munoz, Pablo Pergola, Guillermo E. Umpierrez, Andrea Scalise, Charlie Scott, Robert Lawatscheck, Amer Joseph, George L. Bakris, FIDELIO-DKD and FIGARO-DKD investigators

**Affiliations:** 1 Kidney and Hypertension Unit, Joslin Diabetes Center and Harvard Medical School, Boston, MA; 2 Cardiorenal Translational Laboratory and Hypertension Unit, Institute of Research imas12, CIBER-CV, Hospital Universitario 12 de Octubre, and Faculty of Sport Sciences, European University of Madrid, Madrid, Spain; 3 Department of Cardiology and Berlin Institute of Health Center for Regenerative Therapies, German Centre for Cardiovascular Research Partner Site Berlin, Charité Universitätsmedizin, Berlin, Germany; 4 Institute of Heart Diseases, Wrocław Medical University, Wrocław, Poland; 5 Department of Medicine, University of Michigan School of Medicine, Ann Arbor, MI; 6 Steno Diabetes Center Copenhagen, Herlev, and Department of Clinical Medicine, University of Copenhagen, Copenhagen, Denmark; 7 Clinica de la Costa-Universidad Simon Bolivar, Barranquilla, Colombia; 8 Instituto Nacional de Ciencias Médicas y Nutrición Salvador Zubirán, Mexico; 9 Faculty of Medicine, Universidad de Chile, Department of Nephrology Hospital del Salvador, Santiago, Chile; 10 Colombian College of Hemodynamics and Cardiovascular Intervention, Bogota, Colombia; 11 Renal Associates, PA, San Antonio, TX; 12 Division of Endocrinology, Emory University School of Medicine, Atlanta, GA; 13 Bayer Hispania S.L, Spain; 14 Data Science and Analytics, Bayer PLC, Reading, UK; 15 Clinical Research, Bayer AG, Berlin, Germany; 16 Cardiology and Nephrology Clinical Development, Bayer AG, Berlin, Germany; 17 Department of Medicine, University of Chicago Medicine, Chicago, IL

**Keywords:** Type 2 diabetes, chronic kidney disease, cardiorenal, Hispanic patients, finerenone, nonsteroidal mineralocorticoid receptor antagonist

## Abstract

**Rationale & Objective:**

In FIDELITY, finerenone improved cardiorenal outcomes in patients with chronic kidney disease (CKD) and type 2 diabetes. This analysis explores the efficacy and safety of finerenone in Hispanic patients.

**Study Design:**

Post hoc analysis of the FIDELITY prespecified pooled analysis of the FIDELIO-DKD and FIGARO-DKD randomized control trials.

**Setting & Participants:**

Patients with type 2 diabetes and CKD (urinary albumin-to-creatinine ratio [UACR] of ≥30 to <300 mg/g and estimated glomerular filtration rate [eGFR] of ≥25-≤90 mL/min/1.73 m^2^, or UACR of ≥300 to ≤5,000 and eGFR of ≥25 mL/min/1.73 m^2^) on optimized renin–angiotensin system blockade.

**Intervention:**

Finerenone or placebo.

**Outcomes:**

Cardiovascular composite (cardiovascular death, nonfatal myocardial infarction, nonfatal stroke, or hospitalization for heart failure); kidney composite (kidney failure, sustained ≥57% eGFR decline, or renal death); change in UACR.

**Results:**

Of 13,026 patients, 2,099 (16.1%) self-identified as Hispanic. Median follow-up was 3.0 years. The cardiovascular composite outcome occurred in 10.0% of Hispanic patients receiving Finerenone and in 12.3% of Hispanic patients receiving placebo (HR, 0.80; 95% CI, 0.62-1.04). This was consistent with non-Hispanic patients (HR, 0.87; 95% CI, 0.79-0.97; *P*_interaction_= 0.59). The kidney composite outcome occurred in 6.5% and 6.6% of Hispanic patients with finerenone and placebo, respectively (HR, 0.94; 95% CI, 0.67-1.33). The risk reduction was consistent with that observed in non-Hispanic patients (HR, 0.75; 95% CI, 0.64-0.87; *P*_interaction_= 0.22). Finerenone reduced UACR by 32% at month 4 in both Hispanic and non-Hispanic patients versus placebo (*P* < 0.001 for both patient groups). The safety profile of finerenone and incidence of hyperkalemia was similar between Hispanic and non-Hispanic patient groups.

**Limitations:**

Small sample size, short follow-up time, and lower treatment adherence in the Hispanic population.

**Conclusions:**

Overall, the efficacy and safety of finerenone were similar in Hispanic and non-Hispanic patients with CKD and type 2 diabetes.

**Funding:**

Bayer AG

**Trial Registration:**

ClinicalTrials.gov identifier: NCT02540993, NCT02545049

**Plain-Language Summary:**

Chronic kidney disease (CKD) in patients with type 2 diabetes occurs more frequently in Hispanic patients than in non-Hispanic patients, with a more rapid progression to kidney failure. Treatment with finerenone reduces the risk of having a kidney or heart event (such as starting dialysis or having a heart attack) in patients with CKD and type 2 diabetes. Because clinical trials that investigate treatments for CKD and type 2 diabetes have not included enough Hispanic patients, the benefits of treatments particularly for Hispanic patients are frequently unknown. This study explores the benefits of finerenone in Hispanic patients. Overall, the study shows that finerenone can provide kidney and heart benefits in Hispanic patients with CKD and type 2 diabetes, as it does in non-Hispanic patients.

Diabetes was estimated to affect 537 million adults worldwide in 2021.^1^ Type 2 diabetes (T2D) accounts for >90% of diabetes cases worldwide.^1^ Chronic kidney disease (CKD) affects ∼40% of patients with T2D.^2^ Moreover, patients with CKD are among the highest-risk groups for cardiovascular (CV) events and CV disease.^3^ The prevalence of T2D is disproportionately high in the Hispanic population and one of the highest globally,^4^ affecting 13% of Hispanic adults compared with 8% of non-Hispanic White adults.^5^ Hispanic patients have a higher risk of CKD progression compared with non-Hispanic White patients,^6^ showed by an increased decline in estimated glomerular filtration rate (eGFR)^7^^,^^8^ and high prevalence of albuminuria.^4^ Consequently, the incidence of kidney failure requiring dialysis or transplantation among Hispanic patients is high. Despite a 33% reduction in kidney failure requiring dialysis or transplantation related to diabetes in Hispanic patients living in the United States from 2000-2016, in 2016, the rates in Hispanic patients were still at least 2-fold higher compared with non-Hispanic White patients.^9^ Hispanic patients with diabetes more often develop diabetic complications, including retinopathy,^10^^,^^11^ and have a higher risk of hospitalization requiring intermediate or intensive care compared with non-Hispanic White patients with CKD.^12^ Despite this significant burden of CKD and T2D in the Hispanic population, Hispanic patients are underrepresented in US-based clinical trials,^13^ and few clinical trials have enrolled a sufficient number of Hispanic patients to evaluate treatment effects for CKD and T2D in this population. Indeed, the US Food and Drug Administration is planning to enroll more participants from underrepresented racial and ethnic populations into clinical trials in the United States.^14^

Finerenone is a distinct, selective, nonsteroidal mineralocorticoid receptor antagonist.^15^ The Finerenone in CKD and T2D: Combined FIDELIO-DKD and FIGARO-DKD Trial programme analYsis (FIDELITY) is a prespecified pooled analysis of 2 complementary phase 3 trials, Finerenone in Reducing Kidney Failure and Disease Progression in Diabetic Kidney Disease (FIDELIO-DKD; NCT02540993) and Finerenone in Reducing CV Mortality and Morbidity in Diabetic Kidney Disease (FIGARO-DKD; NCT02545049). In the FIDELITY analysis, finerenone reduced the risk of CKD progression and CV outcomes versus placebo across a broad spectrum of patients with CKD and T2D.^16^ To help address the underrepresentation of Hispanic patients with CKD and T2D and the current lack of treatment effect data in this population, the FIDELITY data set was leveraged to explore responses to treatment by ethnicity. As such, this post hoc analysis evaluated whether the cardiorenal benefits of finerenone observed in the overall population in the FIDELITY analysis are consistent in Hispanic patients with CKD and T2D.

## Methods

### Study Design and Patients

The FIDELITY analysis combines individual patient-level data from the FIDELIO-DKD and FIGARO-DKD phase 3 clinical trials, as described previously and in Table S1.^17^^,^^18^ The trials were conducted in accordance with the principles of the Declaration of Helsinki, and the protocols were approved by relevant regulatory authorities and ethics committees for each trial site; written informed consent was obtained from all patients. Eligible patients were adults aged 18 years or older with CKD and T2D, receiving maximum tolerated labeled doses of a renin–angiotensin system inhibitor and had a serum potassium level of ≤4.8 mmol/L at screening. Patients had either a urinary albumin-to-creatinine ratio (UACR) of ≥30 to <300 mg/g and eGFR of ≥25 to ≤90 mL/min/1.73 m^2^, or a UACR of ≥300 to ≤5,000 mg/g and eGFR of ≥25 mL/min/1.73 m^2^. Standard-of-care therapy as suggested by the Kidney Disease Improving Global Outcomes guidelines (including blood pressure control, glycated hemoglobin [HbA1c] monitoring, and lipid management) was optimized during the run-in period. Patients were asked to self-identify their ethnicity; the Hispanic subgroup included patients who self-identified as Hispanic or Latino ethnicity, and the non-Hispanic subgroup included patients who self-identified as not Hispanic or not Latino.

### Randomization and Masking

Patients were randomly assigned (1:1) to receive double-blind, once-daily oral treatment with finerenone (at titrated doses of 10 or 20 mg) or a matching placebo. Randomization was stratified by region (North America, Europe, Asia, Latin America, and other), albuminuria at screening (UACR of ≥30 to <300 mg/g or ≥300 mg/g), and eGFR at screening (25 to <45, 45 to <60, and ≥60 ml/min/1.73 m^2^). All patients and study personnel (except for the independent data-monitoring committee) were masked from treatment allocation.

### Key Outcomes

Efficacy outcomes included a CV composite outcome of CV death, nonfatal myocardial infarction, nonfatal stroke, or hospitalization for heart failure, and a kidney composite outcome included kidney failure, a sustained decrease of ≥57% in the eGFR from baseline (equivalent to a doubling of the serum creatinine level) maintained for at least 4 weeks, or renal death. Kidney failure was defined as the initiation of long-term dialysis (for ≥90 days) or kidney transplantation or a sustained decrease in eGFR to <15 mL/min/1.73 m^2^. A sustained decline in eGFR required confirmation with a second consecutive central laboratory measurement at least 4 weeks after the initial measurement. Additional efficacy outcomes of interest included changes in UACR and long-term eGFR slope. Safety outcomes and vital signs, such as incidence of adverse events (AEs), hyperkalemia, and change in systolic blood pressure (SBP) were also evaluated. Data for these outcomes are reported for both Hispanic and non-Hispanic patients. All potential efficacy outcomes were prospectively adjudicated by an independent clinical event committee blinded to treatment assignment.

### Statistical Analysis

Efficacy outcomes were analyzed in the pooled analysis set (by intention to treat), comprising all patients randomized who did not have critical Good Clinical Practice violations. Critical Good Clinical Practice violations related to site or patient misconduct resulted in the prospective exclusion of 145 patients (60 patients in FIDELIO-DKD and 85 patients in FIGARO-DKD), leaving a population of 13,026 patients in whom statistical analyses were performed. The included analyses were exploratory. Analyses included descriptive statistics, statistical tests for interaction, subject to a sufficient sample size with a given subgroup, and mixed models for repeated measures. Time-to-event treatment outcomes were expressed as hazard ratios (HRs) with corresponding confidence intervals (CIs). HRs (95% CI) are based on the stratified Cox proportional hazards model estimated within each level of the subgroup variable. The *P* values for interaction between the treatment group (finerenone or placebo) and each baseline subgroup are based on the Cox proportional hazards model, including the terms treatment group, baseline subgroup, and their interaction. The models for the subgroup analyses were stratified by eGFR and albuminuria category at screening, region, history of CV disease, and study (FIDELIO-DKD/FIGARO-DKD). The eGFR was calculated using the CKD Epidemiology Collaboration 2009 equation.^19^ Events were reported from randomization up to the end-of-study visit. The change in UACR was tested with a mixed model, assuming an unstructured covariance matrix and adjusting for treatment, stratification factors, visit, interaction between treatment group and visit, baseline value, and interaction between baseline value and visit. The change in eGFR or eGFR slope (acute slope: baseline to month 4; long-term slope: month 4 to end of study) was evaluated using a 2-slope, linear spline, mixed model for repeated measure analysis.^20^ Unstructured covariance patterns were used across all subgroups. All available eGFR measurements were included in the analyses, irrespective of discontinuation of study treatment.

## Results

### Patients

Of 13,026 patients included in the analysis, 2,099 (16.1%) self-identified as Hispanic; 1,065 (50.7%) received finerenone, and 1,034 (49.3%) received placebo. Overall, 1,368 (65.2%) Hispanic patients were from Latin America, 643 (30.6%) from North America, 67 (3.2%) from Europe, 6 (0.3%) from Asia, and 15 (0.7%) from other regions (Fig S1). Overall, the mean daily dose of study treatment was 16.5 and 17.4 mg for patients randomized to finerenone and placebo, respectively. The mean adherence was 87.8% and 90.3% for Hispanic patients randomized to finerenone and placebo, and 92.5% and 93.2% for non-Hispanic patients, respectively. The median follow-up was 2.6 years for Hispanic patients [interquartile range (IQR), 0.03-4.99 years] and 3.1 years for non-Hispanic patients (IQR, 0.03-5.11 years).

Table 1 displays the baseline characteristics of Hispanic and non-Hispanic patients; baseline characteristics by treatment allocation are provided in Table S2. The Hispanic subgroup included more females (38.4% vs 28.7%) and reported a higher mean eGFR of (60.8 vs 57.0 mL/min/1.73 m^2^), median UACR of (549 vs 507 mg/g), and HbA1c of (8.0% vs 7.6%) than the non-Hispanic patient subgroup. The duration of diabetes was longer in Hispanic patients (16.6 vs 15.2 years), and fewer Hispanic patients were current smokers (9.8% vs 17.3%) compared with non-Hispanic patients. The use of concomitant medications was lower in Hispanic than in non-Hispanic patients, including sodium-glucose co-transporter-2 inhibitors (SGLT-2is; 5.1% vs 7.0%, respectively), glucagon-like peptide-1 receptor agonists (GLP-1Ras; 3.6% vs 8.0%), potassium supplements (1.1% vs 3.3%), and potassium-lowering agents (0.2% vs 1.6%).

**Table 1:** Baseline Demographic and Clinical Characteristics of Hispanic and Non-Hispanic Patients

Characteristic	Hispanic (n=2,099)	Non-Hispanic (n=10,927)
Age, (y), mean ± SD	64.2 ± 9.7	64.9 ± 9.5
Sex, female, n (%)	807 (38.4)	3,131 (28.7)
Systolic blood pressure, mm Hg, mean ± SD	137.0 ± 14.7	136.7 ± 14.1^a^
Diastolic blood pressure, mm Hg, mean ± SD	77.0 ± 9.4	76.2 ± 9.6^b^
BMI, kg/m^2^, mean ± SD	31.3 ± 6.1	31.3 ± 6.0^c^
Waist-to-hip ratio, mean ± SD	1.0 ± 0.2	1.0 ± 0.1^d^
Waist circumference, cm, mean ± SD	105.8 ± 14.4	107.3 ± 15.2^e^
Duration of diabetes, (y), mean ± SD	16.6 ± 9.1	15.2 ± 8.6^f^
HbA1c, (%), mean ± SD	8.0 ± 1.5	7.6 ± 1.3^g^
Serum potassium, mmol/L, mean ± SD	4.39 ± 0.43^b^	4.34 ± 0.44^b^
eGFR, mL/min/1.73 m^2^, mean ± SD	60.8 ± 22.8^b^	57.0 ± 21.4^h^
eGFR, mL/min/1.73 m^2^, n/N (%)^a^		
<45	610 (29.1)	3,784 (34.6)
45 to <60	504 (24.0)	2,930 (26.8)
≥60	983 (46.8)	4,212 (38.5)
UACR, mg/g, median, IQR	549 (249-1,189)^b^	507 (188-1,133)^a^
UACR, mg/g, n (%)^i^		
<30	41 (2.0)	189 (1.7)
30 to <300	576 (27.4)	3,523 (32.2)
≥300	1,480 (70.5)	7,212 (66.0)
Current smoker, n (%)	206 (9.8)	1,887 (17.3)
History of CVD, n (%)	874 (41.6)	5,061 (46.3)
Medication use at baseline, n (%)		
Angiotensin-converting enzyme inhibitors	739 (35.2)	4,340 (39.7)
Angiotensin receptor blockers	1,358 (64.7)	6,579 (60.2)
β-Blockers	930 (44.3)	5,574 (51.0)
Diuretics	999 (47.6)	5,711 (52.3)
Statins	1,489 (70.9)	7,910 (72.4)
Potassium supplements	23 (1.1)	362 (3.3)
Potassium-lowering agents (including binders)	5 (0.2)	177 (1.6)
Glucose-lowering therapies	2,059 (98.1)	10,661 (97.6)
Insulin and analogs	1,301 (62.0)	6,329 (57.9)
Metformin	1,329 (63.3)	6,228 (57.0)
Sulfonylureas	515 (24.5)	2,874 (26.3)
DPP-4 inhibitors	361 (17.2)	2,917 (26.7)
GLP-1RAs	75 (3.6)	869 (8.0)
SGLT-2is	108 (5.1)	769 (7.0)

Abbreviations: BMI, body mass index; CVD, cardiovascular disease; DPP-4, dipeptidyl peptidase-4; eGFR, estimated glomerular filtration rate; GLP-1RA, glucagon-like peptide-1 receptor agonist; HbA1c, glycated hemoglobin; IQR, interquartile range; SD, standard deviation; SGLT-2i, sodium-glucose co-transporter-2 inhibitor; UACR, urinary albumin-to-creatinine ratio

a Missing data for 3 patients.

b Missing data for 2 patients.

c Missing data for 19 patients.

d Missing data for 46 patients.

e Missing data for 34 patients.

f Missing data for 18 patients.

g Missing data for 17 patients.

h Missing data for 1 patient.

i Missing data for 5 patients.

At baseline level, Hispanic patients reported a lower prevalence of comorbidities compared with non-Hispanic patients (Table S3); CV comorbidities included coronary artery disease (23.5% vs 32.1%, respectively) and ischemic stroke (9.1% vs 12.5%), and diabetic complications included diabetic retinopathy (32.1% vs 39.2%) and diabetic neuropathy (22.0% vs 27.8%).

### Efficacy

#### CV Composite Outcome

**Figure 1:**
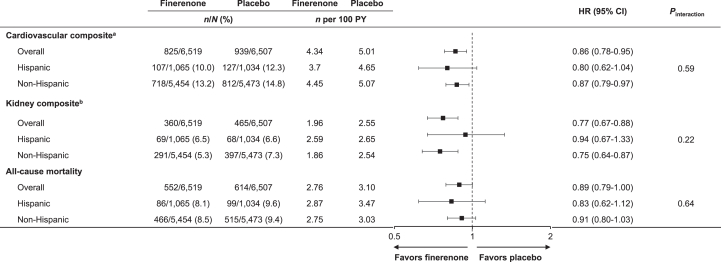
Analysis of cardiovascular and kidney composite outcomes in Hispanic and non-Hispanic patients. ^a^The composite of time to first onset of cardiovascular death, nonfatal myocardial infarction, nonfatal stroke, or hospitalization for heart failure. ^b^The composite of time to first onset of kidney failure, sustained ≥57% decrease in eGFR from baseline over ≥4 weeks, or renal death. CI, confidence interval; eGFR, estimated glomerular filtration rate; HR, hazard ratio; PY, patient-years.

In the overall population, the CV composite outcome occurred in 825 (12.7%) of the 6,519 patients receiving finerenone and in 939 (14.4%) of the 6,507 patients receiving placebo (HR, 0.86; 95% CI, 0.78-0.95). In Hispanic patients, the CV composite outcome occurred in 107 (10%) of the 1,065 patients randomized to finerenone and 127 (12.3%) of the 1,034 patients randomized to placebo (HR, 0.80; 95% CI, 0.62-1.04). This effect was consistent with the risk reduction observed in non-Hispanic patients (HR, 0.87; 95% CI, 0.79-0.97), with no significant interaction between patient subgroups (*P*_interaction_ = 0.59; Fig 1).

#### Kidney Outcomes

In the overall population, the kidney composite outcome occurred in 360 (5.5%) of the 6,519 patients receiving finerenone and in 465 (7.1%) of the 6,507 patients receiving placebo (HR, 0.77; 95% CI, 0.67-0.88). In Hispanic patients, the kidney composite outcome occurred in 69 (6.5%) of the 1,065 patients randomized to finerenone and 68 (6.6%) of the 1,034 patients randomized to placebo (HR, 0.94; 95% CI, 0.67-1.33). This effect was not significantly different from the risk reduction observed in non-Hispanic patients (HR, 0.75; 95% CI, 0.64-0.87), suggesting no heterogeneity in treatment effect between subgroups (*P*_interaction_ = 0.22; Fig 1).

**Figure 2:**
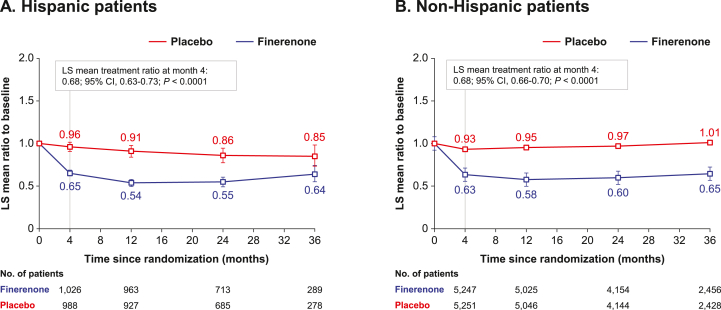
Change in the UACR over time (A) in Hispanic patients and (B) non-Hispanic patients. Mixed model with factors treatment group, region, eGFR category at screening, type of albuminuria at screening, time, treatment∗time, log-transformed baseline value nested within type of albuminuria at screening, and log-transformed baseline value∗time as covariate. CI, confidence interval; eGFR, estimated glomerular filtration rate; LS, least-squares; UACR, urinary albumin-to-creatinine ratio.

In both Hispanic and non-Hispanic patients, finerenone reduced the UACR over time. Finerenone reduced the UACR by 32% at month 4 in both study groups (least-squares [LS] mean treatment ratio, 0.68; 95% CI, 0.63-0.73; *P* < 0.001 and LS mean treatment ratio, 0.68; 95% CI, 0.66-0.70; *P* < 0.001, respectively) compared with placebo (*P*_interaction_ = 0.83; Fig 2).

Finerenone improved the long-term eGFR slope compared with placebo in both Hispanic and non-Hispanic patients over time (Fig S2). In Hispanic patients, the LS mean change in the eGFR from baseline to month 4 was −4.0 mL/min/1.73 m^2^ (95% CI, –4.60 to –3.40) with finerenone and –1.2 mL/min/1.73 m^2^ (95% CI, –1.73 to –0.57) with placebo. In non-Hispanic patients, the LS mean change in the eGFR from baseline to month 4 was –3.2 mL/min/1.73 m^2^ (95% CI, –3.43 to –2.98) with finerenone and –1.0 mL/min/1.73 m^2^ (95% CI, –1.21 to –0.76) with placebo. In Hispanic patients, the long-term eGFR slope from month 4 to end of study was –3.3 mL/min/1.73 m^2^ (95% CI, –3.64 to –2.90) with finerenone and –4.5 mL/min/1.73 m^2^ (95% CI, –4.88 to –4.13) with placebo (LS mean difference, 1.2; 95% CI, 0.71–1.76; *P* < 0.001). In non-Hispanic patients, the long-term eGFR slope from month 4 to end of study was –2.7 mL/min/1.73 m^2^ (95% CI, –2.81 to –2.56) with finerenone and –3.7 mL/min/1.73 m^2^ (95% CI, –3.80 to –3.55) with placebo, (LS mean difference, 0.99; 95% CI, 0.81–1.16; *P* < 0.001).

#### All-Cause Mortality

The effect of finerenone compared with placebo on all-cause mortality was similar in both Hispanic and non-Hispanic patients, with no significant interaction between the patient subgroups (*P*_interaction_ = 0.64; Fig 1).

### Safety and Vital Signs

Fewer treatment-emergent AEs occurred in Hispanic patients than in non-Hispanic patients, irrespective of treatment group (79.9% vs 80.3% of Hispanic patients, and 87.3% vs 87.6% of non-Hispanic patients, in the finerenone and placebo groups, respectively; Table 2). The incidence of serious AEs was numerically lower in Hispanic patients treated with finerenone (19.6%) vs patients treated with placebo (22.6%), and in non-Hispanic patients (34.0% vs 35.8%, respectively).

**Table 2:** Safety Outcomes in Hispanic and Non-Hispanic Patients

Patients with Treatment-Emergent AEs, n (%)	Hispanic Patients		Non-Hispanic Patients	
	Finerenone (n=1,064)	Placebo (n=1,030)	Finerenone (n=5,446)	Placebo (n=5,459)
Any AE	850 (79.9)	827 (80.3)	4,752 (87.3)	4,780 (87.6)
Related to study drug	133 (12.5)	92 (8.9)	1,073 (19.7)	770 (14.1)
Leading to discontinuation	35 (3.3)	42 (4.1)	379 (7.0)	309 (5.7)
Any SAE	209 (19.6)	233 (22.6)	1,851 (34.0)	1,953 (35.8)
Related to study drug	8 (0.8)	3 (0.3)	75 (1.4)	58 (1.1)
Leading to discontinuation	9 (0.8)	19 (1.8)	136 (2.5)	135 (2.5)
Fatal AE	15 (1.4)	21 (2.0)	95 (1.7)	130 (2.4)
Any hyperkalemia	145 (13.6)	86 (8.3)	855 (15.7)	465 (8.5)
Related to study drug	62 (5.8)	35 (3.4)	511 (9.4)	214 (3.9)
Leading to discontinuation	13 (1.2)	6 (0.6)	97 (1.8)	32 (0.6)
Any serious hyperkalemia	7 (0.7)	3 (0.3)	62 (1.1)	13 (0.2)
Leading to hospitalization	7 (0.7)	1 (<0.1)	54 (1.0)	9 (0.2)
Central laboratory assessments				
Serum potassium >5.5 mmol/L	180 (17.3)	94 (9.2)	895 (16.7)	376 (7.0)
Serum potassium >6.0 mmol/L	45 (4.3)	17 (1.7)	166 (3.1)	63 (1.2)

Abbreviations: AE, adverse event; SAE, serious adverse event.

**Figure 3:**
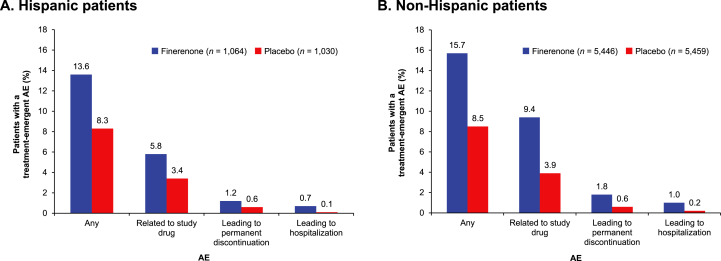
Investigator-reported treatment-emergent AEs related to hyperkalemia (A) in Hispanic patients and (B) non-Hispanic patients. AE, adverse event.

Incidence of treatment-emergent hyperkalemia-related AEs was increased to a similar degree in patients treated with finerenone compared with placebo in both Hispanic and non-Hispanic subgroups (Fig 3); however, incidence of hyperkalemia leading to discontinuation of the study drug was low across both subgroups (Table 2). Increases in serum potassium of >5.5 mmol/L and >6.0 mmol/L were slightly higher in Hispanic patients compared with non-Hispanic patients, irrespective of treatment group (Table 2).

Finerenone had modest effects on SBP compared with placebo; the mean change in SBP was similar in both Hispanic and non-Hispanic patients (Fig S3).

## Discussion

This FIDELITY post hoc analysis shows that finerenone reduced the incidence of cardiorenal events, with no significant treatment differences observed between Hispanic and non-Hispanic patients. Safety outcomes and incidence of hyperkalemia appeared to be similar in both the Hispanic and non-Hispanic subgroups.

Hispanic patients with T2D are at a greater risk for CKD and its progression,^4^, ^5^, ^6^ and social determinants of health are likely to play a role in this.^21^, ^22^, ^23^ Data from the Global Burden of Disease study from 1990 to 2017 shows a negative correlation between socioeconomic status and the burden of CKD associated with T2D, with greater disease-related burden as socioeconomic status declines.^24^ Although the present study lacks data on social determinants of health, such as income, education, and occupation, fewer comorbidities at baseline were observed in Hispanic than in non-Hispanic patients. This may reflect differences in access to and quality of health care, which are often determined by socioeconomic status.^25^ In the present analysis, Hispanic patients were generally similar in age to non-Hispanic patients (64.2 vs 64.9 years, respectively) with a slightly longer duration of diabetes (16.6 vs 15.2 years, respectively), but reported a consistently lower occurrence of CV and diabetic comorbidities. One plausible explanation for this observation is a different level of diagnostics and health care owing to access to care; available evidence suggests an increased risk of undiagnosed medical conditions among racial and ethnic minority populations, including Hispanic patients.^26^ Moreover, Hispanic individuals living in the United States may be less likely than non-Hispanic individuals to receive appropriate diabetes care and to self-monitor their disease^27^ and may be less likely to meet guideline metrics of diabetes care, including HbA1c tests and eye and foot examinations, compared with non-Hispanic White patients.^28^ Therefore, the comorbidity values reported in this analysis may be underestimated compared with their true prevalence. The findings of the current analysis are consistent with the Hispanic health paradox, where for many Hispanic individuals living in the United States, health outcomes seem to be equal to, or better than, outcomes of non-Hispanic White patients despite increased poverty rates, less education, and poorer access to health care.^22^

The racial background of the global Hispanic population is diverse. Approximately 51% of the Brazilian population and 11% of the Colombian population are Afro-descendants, and in a recent study, Hispanic individuals who self-reported as Puerto Rican or Dominican had high levels of African ancestry.^29^^,^^30^ A study showed that African ancestry with 2 copies of the high kidney risk *APOL1* allele are associated with CKD risk in Hispanic patients.^31^ Moreover, the study found that the frequency of eGFR of <60 mL/min/1.73 m^2^ in the Caribbean group (Black, Puerto Rican, or Dominican) was approximately twice that of the Mainland group (Mexican, Central American, or South American background) (4.1% vs 2.1%, respectively; *P* = 5.2 × 10^−9^).^31^ Another study observed faster rates of eGFR decline in Dominican and Puerto Rican individuals compared with non-Hispanic individuals (0.55 and 0.47 mL/min/1.73 m^2^ per year faster, respectively). Mexican, South American, or other Hispanic individuals, whose genetic ancestry is mainly European or Native American, had similar rates of eGFR decline compared with non-Hispanic individuals.^7^^,^^29^ These differences remain statistically significant after adjusting for sociodemographic and clinical factors.

Consistent with previous research,^7^^,^^8^ the rate of eGFR decline was more rapid in Hispanic patients than in non-Hispanic patients despite finerenone treatment (and despite fewer reported comorbidities in the Hispanic patients), thus emphasizing the need for new therapies to reduce the burden of CKD and T2D in Hispanic populations. This observation may be partly explained by a lower adherence to finerenone treatment among Hispanic patients compared with non-Hispanic patients. In addition, use of concomitant SGLT-2i and GLP-1RA treatment was lower in the Hispanic subgroup compared with the non-Hispanic subgroup (SGLT-2i: 5.1% vs 7.0% and GLP-1RA: 3.6% vs 8.0%, respectively), which may have contributed to disparities. However, previous post hoc analyses of the FIDELITY data have shown no significant modification of the effect of finerenone with either SGLT-2i or GLP-1RA treatment at baseline.^32^^,^^33^ However, the long-term eGFR slope was attenuated in patients treated with finerenone irrespective of Hispanic ethnicity, further highlighting the potential benefit of finerenone in this patient population. In the overall population of the FIDELITY analysis, an initial acute eGFR decline was observed shortly after treatment initiation with finerenone in some patients, with a mean change from baseline at month 1 of –2.36 mL/min/1.73 m^2^.^34^ Here, the mean change in eGFR from baseline to month 1 was larger in Hispanic patients (–2.57 mL/min/1.73 m^2^) vs non-Hispanic patients (–2.31 mL/min/1.73 m^2^). Although an acute eGFR decline may raise safety concerns among clinicians, the post hoc analysis of the Reduction of Endpoints in Non–Insulin-Dependent Diabetes Mellitus with the Angiotensin II Antagonist Losartan (RENAAL) trial showed that losartan, an angiotensin II receptor blocker, induced a significantly greater acute eGFR decline during the first 3 months of treatment compared with the placebo but a significantly slower long-term eGFR decline in follow-up.^35^ Similarly, the benefit, efficacy, and safety of SGLT-2is were not modified in a relevant manner by the acute eGFR decline.^36^^,^^37^ Further analyses are needed to understand the efficacy and safety of finerenone in patients experiencing an acute dip in eGFR, particularly in Hispanic patients.

The incidence of potassium levels >5.5 mmol/L was slightly higher in Hispanic patients receiving placebo versus non-Hispanic patients receiving placebo, perhaps because of the tendency toward a higher potassium content in Hispanic diets from a greater consumption of fruits, vegetables, and legumes.^38^^,^^39^ Elevations in serum potassium and the incidence of investigator-reported AEs related to hyperkalemia with finerenone, however, remained similar between the Hispanic and non-Hispanic patient subgroups in the current analysis.

Although this trial enrolled a large number of patients with CKD, the limitations of the current analysis must be acknowledged. A small number of patients self-identified as Hispanic (n=2,099). Subsequently, a low number of events in Hispanic patients were observed (234 CV composite events and 137 kidney composite events). Lack of significant findings among Hispanic patients for the CV and kidney outcomes may therefore be attributable to a lack of power. In addition, the follow-up time was shorter in Hispanic patients compared with non-Hispanic patients. The median study duration was 29 months and 33 months in Hispanic and non-Hispanic patients treated with finerenone, respectively, potentially because of a later recruitment start date in Brazil and Colombia. As previously mentioned, treatment adherence in the finerenone and placebo groups was lower in the Hispanic patients (87.8% and 90.3%, respectively) versus non-Hispanic patients (92.5% and 93.2%, respectively), which has been reported previously,^40^ as was use of concomitant medications. Finally, given the diverse and heterogenous nature of the Hispanic population, particularly in terms of CKD risk factors as highlighted above, the patients selected for inclusion in this study may not be representative of the overall Hispanic population with CKD and T2D.

Hispanic patients are underrepresented in clinical studies that evaluate the cardiorenal outcomes of patients with CKD and T2D. Moreover, the overall FIDELITY analysis showed that it is possible to achieve a diverse representation of patients in clinical trials. This FIDELITY post hoc analysis showed that in both Hispanic and non-Hispanic populations, finerenone is associated with a better overall prognosis of cardiorenal outcomes in patients with T2D at risk of CKD progression. Further research is needed on the socioeconomic and genetic heterogeneity of the Hispanic population to increase our understanding regarding the effectiveness and safety of finerenone.

## Supplementary Material

Supplementary File (PDF) Figure S1-S3; Item S1; Table S1-S3
