# Supplementary material for: Finerenone in Hispanic Patients With CKD and Type 2 Diabetes: A Post Hoc FIDELITY Analysis
Source: Kidney Med. 2023 Aug 1;5(10):100704. doi: 10.1016/j.xkme.2023.100704 (PMC10514441; doi:10.1016/j.xkme.2023.100704)
Supplement: Supplementary File (PDF) — Figure S1-S3; Item S1; Table S1-S3 [file mmc2.docx]

Finerenone in Hispanic Patients With Chronic Kidney Disease and Type 2 Diabetes: A Post Hoc FIDELITY Analysis

**Supplemental Material**

[Participating countries and investigators 2](#_Toc130900927)

[Supplemental Table 1. FIDELIO-DKD and FIGARO-DKD study details as published in the FIDELITY analysis 10](#_Toc130900928)

[Supplemental Table 2. Baseline Demographic and Clinical Characteristics of Hispanic and Non-Hispanic Patients by Treatment Allocation 12](#_Toc130900929)

[Supplemental Table 3. Medical History Findings of Interest at Baseline by Treatment Allocation 15](#_Toc130900930)

[Supplemental Figure 1. Distribution of randomized subjects in the Hispanic subgroup.. 17](#_Toc130900931)

[Supplemental Figure 2. Change in eGFR over time in Hispanic and non-Hispanic patients 18](#_Toc130900932)

[Supplemental Figure 3. Mean change in SBP over time in Hispanic and non-Hispanic patients 19](#_Toc130900933)

Participating Countries and Investigators

**Argentina:** Diego Aizenberg, Inés Bartolacci, Diego Besada, Julio Bittar, Mariano Chahin, Alicia Elbert, Elizabeth Gelersztein, Alberto Liberman, Laura Maffei, Federico Pérez Manghi, Hugo Sanabria, Augusto Vallejos, Gloria Viñes, Alfredo Wassermann

**Australia**: Walter Abhayaratna, Shamasunder Acharya, Peter Colman, David Colquhoun, Chris Ellis, Kim Joshua, Elif Ekinci, Darren Lee, Richard MacIsaac, Peak Mann Mah, Craig Nelson, David Packham, Alexia Pape, Elizabeth Vale Eugenia Pedagogos, Paul Regal, Simon Roger, Hugo Stephenson, Michael Suranyi, Duncan Topliss, James Vandeleur, Johan Verjans, Gary Wittert, Katie-Jane Wynne

**Austria:** Martin Clodi, Christoph Ebenbichler, Evelyn Fliesser-Görzer, Ursula Hanusch, Michael Krebs, Karl Lhotta, Bernhard Ludvik, Gert Mayer, Peter Neudorfer, Bernhard Paulweber, Rudolf Prager, Wolfgang Preiß, Friedrich Prischl, Gerit-Holger Schernthaner, Harald Sourij, Martin Wiesholzer, Heinz Drexel, Rainer Oberbauer, Hans‑Robert Schönherr

**Belgium**: Peter Doubel, Wendy Engelen, Pieter Gillard, Jean-Michel Hougardy, Jean-Marie Krzesinski, Bart Maes, Marijn Speeckaert, Koen Stas, Luc van Gaal, Hilde Vanbelleghem, Francis Duyck, André Scheen

**Brazil:** Daniela Antunes, Roberto Botelho, Claudia Brito, Luis Canani, Maria Eugenia Canziani, Maria Cerqueira, Rogerio de Paula, Freddy Eliaschewitz, Carlos Eduardo Figueiredo, Adriana Forti, Miguel Hissa, Maurilo Leite Jr, Emerson Lima, Irene Noronha, Bruno Paolino, Nathalia Paschoalin, Raphael Paschoalin, Roberto Pecoits Filho, Marcio Pereira, Evandro Portes, Dalton Precoma, Rosangela Rea, Miguel Riella, Joao Eduardo Salles, Eduardo Vasconcellos, Sergio Vencio, Marcelo Bacci, Lilia Maia, Aline Villacorta

**Bulgaria:** Emiliya Apostolova, Radostina Boshnyashka, Ghassan Farah, Dimitar Georgiev, Valentina Gushterova, Neli Klyuchkova, Mariya Lucheva, Petya Manova, Dotska Minkova, Boyan Nonchev, Mariyana Pichmanova, Zhulieta Prakova, Rangel Rangelov, Rosen Rashkov, Pavel Stanchev, Bilyana Stoyanovska-Elencheva, Zhivko Tagarev, Theodora Temelkova-Kurktschieva, Svetla Vasileva, Mariana Yoncheva-Mihaylova, Angel Marinchev, Mariya Miteva

**Canada:** Paul Barre, Brian Carlson, James Conway, Serge Cournoyer, Richard Dumas, Sameh Fikry, Richard Goluch, Pavel Hamet, Randolph Hart, Sam Henein, Joanne Liutkus, Francois Madore, Valdemar Martinho, Giuseppe Mazza, Philip McFarlane, Dennis O’ Keefe, Sean Peterson, Daniel Schwartz, Daniel Shu, Andrew Steele, Guy Tellier, Karthik Tennankore, Sheldon Tobe, George Tsoukas, Richard Tytus, Louise Vitou, Michael Walsh, Stanley Weisnagel, Igor Wilderman, Jean-Francois Yale, Fadia El Boreky, Alan Kelly, Lawrence Leiter, Ivor Teitelbaum

**Chile:** Jorge Cobos, Juan Godoy, Fernando González, Sergio Lobos, Juan Carlos Palma, Juan Carlos Prieto Dominguez, Eliana Reyes, Carmen Romero, Victor Saavedra, Mario Vega, Marcelo Medina, Paola Varleta

**China:** Ruifang Bu, Hanqing Cai, Nan Chen, Qinkai Chen, Dejun Chen, Jinluo Cheng, Youping Dong, Junwu Dong, Tianjun Guan, Chuanming Hao, Wen Huang, Fangfang Jiang, Minxiang Lei, Ling Li, Zhonghe Li, Xuemei Li, Jingmei Li, Yan Li, Xinling Liang, Bo Liang, Fang Liu, Yinghong Liu, Yuantao Liu, Zhihong Liu, Gang Long, Guoyuan Lu, Weiping Lu, Yibing Lu, Ping Luo, Jianhua Ma, Zhaohui Mo, Jianying Niu, Ai Peng, Jiansong Shen, Feixia Shen, Bingyin Shi, Qing Su, Zhuxing Sun, Shuifu Tang, Nanwei Tong, Hao Wang, Xinjun Wang, Guixia Wang, Jianqin Wang, Yangang Wang, Li Wang, Jiali Wei, Tianfeng Wu, Chaoqing Wu, Changying Xing, Fei Xiong, Xudong Xu, Ning Xu, Tiekun Yan, Jinkui Yang, Aiping Yin, Longyi Zeng, Hao Zhang, Yanlin Zhang, Ying Zhang, Wenjing Zhao, Zhiquan Zhao, Hongguang Zheng, Ling Zhong, Dalong Zhu, Yongze Zhuang, Yuming Du, Yi Fang, Weiying Guo, Sheng Jiang, Jian Kuang, Dongmei Li, Hongmei Li, Yinan Li, Yuxiu Li, Jian Liu, Yu Liu, Heng Miao, Wen Peng, Lihua Wang, Mingtong Xu, Liyong Zhong, Jun Zhu

**Colombia:** Clara Arango, Sandra Barrera, Nelly Beltrán López, Diego Benitez, Guillermo Blanco, Andrés Cadena, Julian Coronel, Carlos Cure, Carlos Durán, Alexander González, Gustavo Guzmán, Eric Hernández, Jaime Ibarra, Carlos Jaramillo, Nicolás Jaramillo, William Kattah, Dora Molina, Gregorio Sánchez, Mónica Terront, Freddy Trujillo, Miguel Urina, Ruben Vargas, Iván Villegas, Hernán Yupanqui, Edgar Arcos, Gustavo Aroca, Germán Barreto, Andres Bermudez, Rodrigo Botero, Tatiana Cárdenas, Wilmer Figueroa, Mónica Jaramillo, Manuel Liévano, Mónica López, Dora Molina, Ricardo Rosero, Pedro Trillos

**Czech Republic:** Dino Alferi, Michal Brada, Jiri Brezina, Petr Bucek, Tomas Edelsberger, Drahomira Gulakova, Jitka Hasalova Zapletalova, Olga Hola, Lucie Hornova, Jana Houdova, Helena Hrmova, David Karasek, Sarka Kopecka, Richard Kovar, Eva Krcova, Jiri Kuchar, Vlasta Kutejova, Hana Lubanda, Ivo Matyasek, Magdalena Mokrejsova, Libor Okenka, Martin Prazny, Jiri Pumprla, Pavel Tomanek

**Denmark:** Ulla Andersen, Alin Andries, Jesper Bech, Jens Faber, Gunnar Gislason, Jørgen Hangaard, Grzegorz Jaroslaw Pacyk, Claus Juhl, Thure Krarup, Morten Lindhardt, Sten Madsbad, Joan Nielsen, Ulrik Pedersen-Bjergaard, Per Poulsen, Ole Rasmussen, Peter Rossing, Karoline Schousboe, Jeppe Gram, Thomas Lauridsen, Erling Pedersen, Birger Thorsteinsson

**Finland:** Päivi Flöjt, Mikko Honkasalo, Mikko Honkasalo, Kari Humaloja, Kristiina Kananen, Ilkka Kantola, Arvo Koistinen, Pirkko Korsoff, Jorma Lahtela, Sakari Nieminen, Tuomo Nieminen, Karita Sadeharju, Jorma Strand, Sakari Sulosaari

**France:** Bertrand Cariou, François Chantrel, Sylvaine Clavel, Christian Combe, Jean-Pierre Fauvel, Karim Gallouj, Didier Gouet, Bruno Guerci, Dominique Guerrot, Maryvonne Hourmant, Alexandre Klein, Christophe Mariat, Michel Marre, Rafik Mesbah, Yannick Le Meur, Arnaud Monier, Olivier Moranne, Ronan Roussel, Pierre Serusclat, Benoit Vendrely, Bruno Verges, Philippe Zaoui

**Germany:** Christoph Axthelm, Andreas Bergmann, Andreas L. Birkenfeld, Hermann Braun, Klaus Busch, Christel Contzen, Stefan Degenhardt, Karl Derwahl, Thomas Giebel, Andreas Hagenow, Hermann Haller, Christoph Hasslacher, Thomas Horacek, Wolfgang Jungmair, Christof Kloos, Thorsten Koch, Thilo Krüger, Anja Mühlfeld, Joachim Müller, Andreas Pfützner, Frank Pistrosch, Andrea Rinke, Ludger Rose, Lars Rump, Volker Schettler, Ingolf Schiefke, Heike Schlichthaar, Bernd Schröppel, Norbert Schöll, Kristin Schubert, Thomas Schürholz, Helena Sigal, Lutz Stemler, Georg Strack, Heidrun Täschner, Nicole Toursarkissian, Diethelm Tschöpe, Achim Ulmer, Markus van der Giet, Christoph Wanner, Bernhard R. Winkelmann

**Greece:** Ioannis Boletis, George Dimitriadis, Erifili Hatziagelaki, Christos Iatrou, Ioannis Ioannidis, Theodora Kounadi, Ioanna Makriniotou, Dorothea Papadopoulou, Aikaterini Papagianni, Ploumis Passadakis, George Piaditis, Ioannis Stefanidis

**Hong Kong:** Tai Pang Ip, Paul Lee, On Yan Andrea Luk, Ronald Ma, Wing Sun Chow, Angela Wang, Vincent Yeung

**Hungary:** Dora Bajcsi, Peter Danos, Eleonora Harcsa, Akos Kalina, Szilvia Kazup, Katalin Keltai, Robert Kirschner, Julianna Kiss, Laszlo Kovacs, Beata Lamboy, Botond Literati-Nagy, Margit Mileder, Laszlo Nagy, Ebrahim Noori, Gabor Nyirati, Gizella Petro, Karoly Schneider, Judit Simon, Albert Szocs, Szilard Vasas, Krisztina Wudi, Zsolt Zilahi, Marianna Zsom

**Ireland:** Joe Eustace, John Holian, Donal Reddan, Yvonne O’ Meara

**Israel:** Rosane Abramof Ness, Faiad Adawi, Zaher Armaly, Shaul Atar, Amir Bashkin, Sydney Ben Chetrit, Noa Berar Yanay, Gil Chernin, Mahmud Darawsha, Shai Efrati, Mazen Elias, Evgeny Farber, Mariela Glandt, Ehud Grossman, Majdi Halabi, Ilana Harman-Boehm, Khaled Khazim, Idit Liberty, Oscar Minuchin, Ofri Mosenzon, Farid Nakhoul, Assy Nimer, Doron Schwartz, Julio Wainstein, Yoram Yagil, Robert Zukermann

**Italy:** Angelo Avogaro, Giovanni Giorgio Battaglia, Maurizio Tiziano Bevilacqua, Enzo Bonora, Carlo Antonio Bossi, Paolo Calabrò, Franco Luigi Cavalot, Roberto Cimino, Mario Gennaro Cozzolino, Salvatore David, Michele Emdin, Enrico Fiaccadori, Paolo Fiorina, Carlo Bruno Giorda, Maria Cristina Gregorini, Gaetano La Manna, Davide Carlo Maggi, Roberta Manti, Giancarla Meregalli, Antonello Pani, Aneliy Ilieva Parvanova, Norberto Perico, PierMarco Piatti, Antonio Pisani, Antonio Ettore Pontiroli, Paola Ponzani, Gennaro Santorelli, Domenico Santoro, Renzo Scanziani, Ugo Teatini, Giancarlo Tonolo, Roberto Trevisan, Anna Maria Veronelli, Giorgio Luciano Viviani

**Japan:** Hideo Araki, Yukihiro Bando, Osamu Ebisui, Naruhiro Fujita, Hirotaka Fukasawa, Ryuichi Furuya, Yoshiyuki Hamamoto, Akihiro Hamasaki, Kotaro Hasegawa, Masahiro Hatazaki, Terumasa Hayashi, Takayuki Higashi, Yoshihide Hirohata, Shuji Horinouchi, Ayumu Hoshi, Hirofumi Imoto, Akemi Inagaki, Masayuki Inagaki, Daijo Inaguma, Toshihiko Inoue, Masao Ishii, Tamayo Ishiko, Motohide Isono, Hideaki Jinnouchi, Hidetoshi Kanai, Daisuke Kanda, Hideo Kanehara, Masayuki Kashima, Yuko Kataoka, Shigehiro Katayama, Kiyoe Kato, Takeshi Katsuki, Katsunori Kawamitsu, Satsuki Kawasaki, Fumi Kikuchi, Hidetoshi Kikuchi, Rui Kishimoto, Kunihisa Kobayashi, Junko Koide, Rieko Komi, Miyuki Kubota, Genpei Kuriya, Takeshi Kurose, Yoshiro Kusano, Hajime Maeda, Sunao Matsubayashi, Kazunari Matsumoto, Naoya Matsumura, Yasuto Matsuo, Naoki Matsuoka, Hiroaki Miyaoka, Satoshi Miyata, Takeshi Morita, Isao Murakami, Satoshi Murao, Udai Nakamura, Mikihiro Nakayama, Jun Nakazawa, Sakae Nohara, Takashi Nomiyama, Masayuki Noritake, Yoshiaki Oda, Takayuki Ogiwara, Hiroshi Ohashi, Hideki Okamoto, Shinichi Okino, Takeshi Osonoi, Nobuhiro Sasaki, Yoshitaka Sayo, Taiji Sekigami, Taro Shibasaki, Hirotaka Shibata, Tatsushi Shimoyama, Junji Shinoda, Hiroshi Sobajima, Kazuya Sugitatsu, Toshiyuki Sugiura, Toru Sugiyama, Daisuke Suzuki, Hiroyuki Suzuki, Masaaki Suzuki, Asami Takeda, Asami Tanaka, Seiichi Tanaka, Izumi Tsunematsu, Yasuo Ueda, Soichi Uekihara, Makoto Ujihara, Ken Yajima, Daishiro Yamada, Masayo Yamada, Kazuo Yamagata, Ken Yamakawa, Fumiko Yamakawa, Yoshimitsu Yamasaki, Yuko Yambe, Taihei Yanagida, Hidekatsu Yanai, Toshihiko Yanase, Tetsuyuki Yasuda

**Lithuania:** Dovile Kriauciuniene, Jurate Lasiene, Antanas Navickas, Lina Radzeviciene, Egle Urbanaviciene, Gediminas Urbonas, Audrone Velaviciene

**Malaysia:** Rohana Abd Ghani, Nor Azizah Aziz, Li Yuan Lee, Chek Loong Loh, Norhaliza Mohd Ali, Nurain Mohd Noor, Nik Nur Fatnoon Nik Ahmad, Jeyakantha Ratnasingam, Wan Hasnul Halimi Bin Wan Hasan, Wan Mohd Izani Wan Mohamed, Rizmy Najme Khir, Masni Mohamad, Tong Boon Alexander Tan

**Mexico:** Sandro Avila Pardo, Miriam Bastidas Adrian, Alfredo Chew Wong, Jorge Escobedo de la Peña, Guillermo Fanghänel Salmón, Guillermo González Gálvez, Ramiro Gutiérrez Ochoa, Saúl Irizar Santana, Magdalena Madero Rovalo, Gustavo Méndez Machado, Luis Nevarez Ruiz, Denisse Ramos Ibarra, Gabriel Ramos López, Leobardo Sauque Reyna, Gustavo Solache Ortiz, Rafael Valdez Ortiz, Juan Villagordoa Mesa, Melchor Alpizar Salazar, Pedro García Hernández, José González, José Lazcano Soto, Arturo Saldaña Mendoza, Sergio Irizar Santana, Elvira González Vilchis

**Netherlands:** R.C. Bakker, J.N.M. Barendregt, A.H. Boonstra, Willem Bos, C.B. Brouwer, M. van Buren, Ron Gansevoort, Adriaan Kooy, Marielle Krekels, Ruud J.M. van Leendert, Louis A.G. Lieverse, P.T. Luik, E. Lars Penne, Peter Smak Gregoor, Liffert Vogt, Bert-Jan van den Born

**New Zealand:** John Baker, Veronica Crawford, Rick Cutfield, Peter Dunn, Jeremy Krebs, Kingsley Nirmalaraj, Russell Scott, Nine Smuts, Janet Titchener

**Norway:** Erik Eriksen, Trine Finnes, Hans Høivik, Thomas Karlsson, Peter Scott Munk, Maria Radtke, Knut Risberg, Jan Rocke, Leidulv Solnør, Aud-Eldrid Stenehjem, Anne-Beathe Tafjord, Emil Asprusten, Robert Hagemeier, Kjetil Høye, Hilde Selsås, Frode Thorup, Cecilie Wium

**Philippines:** Glenda Pamugas, Araceli Panelo, Ronald Perez, Maribel Tanque, Louie Tirador, Michael Villa, Albert Bautista, Elizabeth Catindig, Carlo Manalo, Roberto Mirasol

**Poland:** Patrycja Butrymowicz, Kazimierz Ciechanowski, Grazyna Cieslik, Edward Franek, Janusz Gumprecht, Michal Hoffmann, Jolanta Krzykowska, Ilona Kurnatowska, Katarzyna Landa, Adam Madrzejewski, Katarzyna Madziarska, Stanislaw Mazur, Piotr Napora, Michal Nowicki, Anna Ocicka-Kozakiewicz, Barbara Rewerska, Teresa Rusicka, Jan Ruxer, Ewa Skokowska, Andrzej Stankiewicz, Tomasz Stompor, Agnieszka Tiuryn-Petrulewicz, Katarzyna Wasilewska, Bogna Wierusz-Wysocka, Renata Wnetrzak-Michalska, Krystyna Jedynasty, Izabela Sein Anand

**Portugal:** Edgar Almeida, Rosa Ballesteros, Carlos Barreto, Idalina Beirao, Rita Birne, Cesar Esteves, Jose Guia, Susana Heitor, Olinda Marques, Pedro Melo, Fernando Nolasco, Amalia Pereira, Cristina Roque, Francisco Rosario, Gil Silva, Ana Silva, Fernando Teixeira e Costa, Ana Vila Lobos, Ana Rita Alves, Ilidio Brandao, Rui Carvalho, Joao Coelho, Ana Lourenco, Pedro Matos, Vanisa Rosario, Joao Sergio Neves

**Puerto Rico:** Gregorio Cortes-Maisonet, Amaury Roman-Miranda, Yudit Brito-Peguero, Gildred Colon-Vega

**Romania:** Adrian Albota, Cornelia Bala, Hortensia Barbonta, Elena Caceaune, Doina Catrinoui, Ciprian Constantin, Adriana Dumitrescu, Nicoleta Mindrescu, Cristina Mistode, Gabriela Negrisanu, Adriana Onaca, Silvia Paveliu, Ella Pintilei, Lavinia Pop, Amorin Popa, Alexandrina Popescu, Gabriela Radulian, Iosif Szilagyi, Liana Turcu, Georgeta Vacaru, Adrian Vlad, Adriana Filimon, Ioan Veresiu

**Russia:** Mikhail Antsiferov, Mikhail Arkhipov, Andrey Babkin, Olga Barbarash, Vitaliy Baranov, Elena Chernyavskaya, Arkadiy Demko, Alexander Dreval, Anton Edin, Polina Ermakova, Valentin Fadeev, Albert Galyavich, Leyla Gaysina, Ivan Gordeev, Irina Ipatko, Marina Kalashnikova, Yuriy Khalimov, Vadim Klimontov, Zhanna Kobalava, Elena Kosmacheva, Natalya Koziolova, Lyudmila Kvitkova Sergey Levashov, Roman Libis, Vyacheslav Marasaev, Natalia Malykh, Vladimir Martynenko, Sofya Malyutina, Imad Merai, Ashot Mkrtumyan, Galina Nechaeva, Nina Petunina, Shamil Palyutin, Leonid Pimenov, Elena Rechkova, Tatyana Rodionova, Oksana Rymar, Ruslan Sardinov, Olga Semenova, Alexander Sherenkov, Oleg Solovev, Elena Smolyarchuk, Leonid Strongin, Olga Ukhanova, Nadezhda Verlan, Natalya Vorokhobina, Davyd Yakhontov, Sergey Yakushin, Elena Zakharova, Alsu Zalevskaya, Olga Zanozina, Elena Zhdanova, Larisa Zhukova, Tatyana Zykova, Yulia Argunova, Konstantin Nikolaev, Svetlana Villevalde

**Singapore:** Chee Fang Sum, Sufi Muhummad Suhail, Ru San Tan, Anantharaman Vathsala, Edmund Wong, Yong Mong Bee

**Slovakia:** Jana Babikova, Ingrid Buganova, Andrej Dzupina, Zuzana Ochodnicka, Dalibor Sosovec, Denisa Spodniakova, Peter Minarik

**South Africa:** Fayzal Ahmed, Aslam Amod, Sindeep Bhana, Larry Distiller, Dirkie Jansen van Rensburg, Mukesh Joshi, Shaifali Joshi, Deepak Lakha, Essack Mitha, Gracjan Podgorski, Naresh Ranjith, Brian Rayner, Paul Rheeder, Mohamed Sarvan, Mary Seeber, Heidi Siebert, Mohammed Tayob, Julien Trokis, Dorothea Urbach, Louis van Zyl, Dirkie Jansen van Rensburg

**South Korea:** Bum-Soon Choi, Moon Gi Choi, ChoonHee Chung, YouCheol Hwang, ChongHwa Kim, InJoo Kim, JaeHyeon Kim, SinGon Kim, SungGyun Kim, Tae Hee Kim, WooJe Lee, ByungWan Lee, Kang Wook Lee, Kook-Hwan Oh, Ji Eun Oh, Yun Kyu Oh, Dong-Jin Oh, Junbeom Park, Seok Joon Shin, Su-Ah Sung, Jae Myung Yu, HyeSoo Chung, Ji Hye Huh, JunGoo Kang, ChulSik Kim, HyeSoon Kim, NamHoon Kim, Soo Lim, Young Min Cho, Cheol Young Park

**Spain:** Irene Agraz, Francisco Javier Ampudia, Hanane Bouarich, Francesca Calero, Cristina Castro, Secundino Cigarrán Guldris, Josep Cruzado Garrit, Fernando de Álvaro, Josep Galcerán, Olga González Albarrán, Julio Hernández Jaras, Meritxell Ibernón, Francisco Martínez Deben, Mª Dolores Martínez Esteban, José María Pascual Izuel, Judith Martins, Juan Mediavilla, Alfredo Michán, Julio Pascual Santos, Esteban Poch, Manuel Polaina Rusillo, Carlos Sánchez Juan, Rafael Santamaría Olmo, José Julián Segura de la Morena, Alfonso Soto, Maribel Troya, Fernando Cereto Castro, Pablo Gómez Fernández, Laura Fuentes Sánchez, Mercedes González Moya, Domingo Hernández Marrero, Gonzalo Piedrola Maroto, Josep Redón, Daniel Seron

**Sweden:** Annette Bruchfeld, Dan Curiac, Ken Eliasson, Malin Frank, Gregor Guron, Olof Hellberg, Margareta Hellgren, Hans Larnefeldt, Carl-Johan Lindholm, Magnus Löndahl, Erik Rein-Hedin, Inga Soveri, Jonas Spaak, Bengt-Olov Tengmark, Cornelia Lif-Tiberg, Johan Månflod, Han Nguyen

**Switzerland:** Daniel Ackermann, Stefan Bilz, Michel Burnier, Christian Forster, Stefan Kalbermatter, Andreas Kistler, Antoinette Pechère-Bertschi, Bernd Schultes, Markus Laimer, Gottfried Rudofsky, Christopher Strey, Gregoire Wuerzner

**Taiwan:** Chiz-Tzung Chang, Cheng-Chieh Hung, Ju-Ying Jiang, Chien-Te Lee, Shuei-Liong Lin, Der-Cherng Tarng, Shih-Te Tu, Mai-Szu Wu, Ming-Ju Wu, Lee-Ming Chuang

**Thailand:** Chaicharn Deerochanawong, Chagriya Kitiyakara, Vuddhidej Ophascharoensuk, Chatlert Pongchaiyakul, Bancha Satirapoj, Natapong Kosachunhanan, Piyamitr Sritara

**Turkey:** Necmi Eren, Ibrahim Gul, Okan Gulel, Ismail Kocyigit, Abdulbaki Kumbasar, Idris Sahin, Ramazan Sari, Burak Sayin, Talat Tavli, Sedat Ustundag, Yavuz Yenicerioglu, Ozer Badak, Murat Cayli, Aytekin Oguz, Oner Ozdogan, Ibrahim Sari, Ahmet Temizhan, Mustafa Tigen, Ugur Turk, Huseyin Yilmaz, Mehmet Yilmaz

**Ukraine:** Iryna Bondarets, Volodymyr Botsyurko, Viktoriia Chernikova, Oleksandra Donets, Ivan Fushtey, Mariia Grachova, Anna Isayeva, Dmytro Kogut, Julia Komisarenko, Nonna Kravchun, Kateryna Malyar, Borys Mankovsky, Liliya Martynyuk, Vitaliy Maslyanko, Halyna Myshanych, Larysa Pererva, Nataliia Pertseva, Oleksandr Serhiyenko, Ivan Smirnov, Liubov Sokolova, Vasyl Stryzhak, Maryna Vlasenko, Ganna Isayeva, Oleksandr Larin

**United Kingdom:** Ahmad AbouSaleh, Jonathan Barratt, Cuong Dang, Hassan Kahal, Adam Kirk, Anne Kilvert, Sui Phi Kon, Kieran McCafferty, Dipesh Patel, Sam Rice, Arutchelvam Vijayaraman, Yuk-ki Wong, Martin Gibson, Mona Wahba, Reza Zaidi, Rudy Bilous, Andrew Johnson, Dhanya Kalathil, Anne Kilvert, Christina Kyriakidou, Amit Mathew, Rasha Mukhtar, Imrozia Munsoor, Anton Poterajlo, Pauline Swift

**United States:** Idalia Acosta, Atoya Adams, Sharon Adler, Dilawar Ajani, Slamat Ali, Radica Alicic, Amer Al-Karadsheh, Sreedhara Alla, D. Allison, Nabil Andrawis, Ahmed Arif, Ahmed Awad, Masoud Azizad, Michael Bahrami, Shweta Bansal, Steven Barag, Ahmad Barakzoy, Mark Barney, Joshua Barzilay, Khalid Bashir, Jose Bautista, Srinivasan Beddhu, Diogo Belo, Sabrina Benjamin, Ramin Berenji, Anuj Bhargava, Jose Birriel, Stephen Brietzke, Frank Brosius, Osvaldo Brusco, Anna Burgner, Robert Busch, Rafael Canadas, Maria Caramori, Jose Cardona, Christopher Case, Humberto Cruz, Ramprasad Dandillaya, Dalia Dawoud, Zia Din, Bradley Dixon, Ankur Doshi, James Drakakis, Mahfouz El Shahawy, Ashraf El-Meanawy, Mohammed El-Shahawy, John Evans, George Fadda, Umar Farooq, Roland Fernando, Raymond Fink, Brian First, David Fitz-Patrick, John Flack, Patrick Fluck, Leon Fogelfeld, Vivian Fonseca, Juan Frias, Claude Galphin, Luis Garcia-Mayol, Gary Goldstein, Edgar Gonzalez, Francisco Gonzalez-Abreu, Ashwini Gore, David Grant, Violet Habwe, Maxine Hamilton, Jamal Hammoud, Stuart Handelsman, Israel Hartman, Glenn Heigerick, Andrew Henry, German Hernandez, Carlos Hernandez-Cassis, Carlos Herrera, Joachim Hertel, Wenyu Huang, Rogelio Iglesias, Ali Iranmanesh, Timothy Jackson, Mahendra Jain, Kenneth Jamerson, Karen Johnson, Eric Judd, Joshua Kaplan, Zeid Kayali, Bobby Khan, Muhammad Khan, Sourabh Kharait, M. Sue Kirkman, Nelson Kopyt, Wayne Kotzker, Csaba Kovesdy, Camil Kreit, Arvind Krishna, Saeed Kronfli, Keung Lee, Derek LeJeune, Brenda Lemus, Carlos Leon-Forero, Douglas Linfert, Henry Lora, Alexander Lurie, Geetha Maddukuri, Alexander Magno, Louis Maletz, Sreedhar Mandayam, Mariana Markell, Ronald Mayfield, Caroline Mbogua, Dierdre McMullen, Carl Meisner, Stephen Minton, Bharat Mocherla, Rajesh Mohandas, Manuel Montero, Moustafa Moustafa, Salil Nadkarni, Samer Nakhle, Jesus Navarro, Nilda Neyra, Romanita Nica, Philip Nicol, Paul Norwood, Visal Numrungroad, Richard O’ Donovan, A. Odugbesan, Jorge Paoli-Bruno, Samir Parikh, Rakesh Patel, Aldo Peixoto, Pablo Pergola, Alan Perlman, Karlton Pettis, Roberto Pisoni, Mirela Ponduchi, Jorge Posada, Sharma Prabhakar, Jai Radhakrishnan, Mahboob Rahman, Rupesh Raina, Anjay Rastogi, Efrain Reisin, Marc Rendell, David Robertson, Michael Rocco, Hugo Romeu, Sylvia Rosas, Jack Rosenfeld, Dennis Ross, Jeffrey Rothman, Lance Rudolph, Yusuf Ruhullah, Gary Ruoff, Jeffrey Ryu, Mandeep Sahani, Ramin Sam, Garfield Samuels, William Sanchez, Vladimir Santos, Scott Satko, Sanjeev Saxena, David Scott, Gilberto Seco, Melvin Seek, Harvey Serota, Tariq Shafi, Nauman Shahid, Michael Shanik, Santosh Sharma, Arjun Sinha, James Smelser, Mark Smith, Kyaw Soe, Richard Solomon, Eugene Soroka, Joseph Soufer, Bruce Spinowitz, Leslie Spry, Rosa Suarez, Bala Subramanian, Harold Szerlip, Aparna Tamirisa, Stephen Thomson, Tuan-Huy Tran, Richard Treger, Gretel Trullenque, Thomas Turk, Guillermo Umpierrez, Daniel Urbach, Martin Valdes, Shujauddin Valika, Damaris Vega, Peter Weissman, Adam Whaley-Connell, Jonathan Winston, Jonathan Wise, Alan Wynne, Steven Zeig, Emaad Abdel-Rahman, Edel Abreu, Alaa Awad, Nader Bahri, John Bertsch, David Bleich, Jonathan Bornfreund, Harjeet Brar, Susan Brian, Cynthia Brinson, Humberto Bruschetta, Jose Carpio, Steven Cohen, John Cosby, Soni Dhanireddy, Jorge Diaz, Fredrick Dunn, Sabitha Eppanapally, Joseph Fayad, Archana Goel, Kanakadurga Govindaraju, Stephen Halpern, Audrey Jones, William Kaye, Herbert Knight, Stanley Koch, Nandini Kohli, Guido Lastra, Sam Lerman, Jorge Loredo, Dragana Lovre, Mustafa Mandviwala, Earl Martin, Jill Meyer, John Murray, David Oliver, Suzanne Oparil, Jesus Penabad, Isabel Pereira, Larry Popeil, Gonzalo Quesada, Kodangudi Ramanathan, Luis Ramos-Gonez, Mandana Rastegar, Padmashri Rastogi, Juan Rondon, Prabir Roy-Chaudhury, David Smith, Don Williamson, Catherine Womack, Hala Yamout, Michael Yuryev

**Vietnam:** Phuong Chu, Lam Van Hoang, Tran Khanh, Nguyen Thi Phi Nga, Pham Nguyen Son, Lan Phuong Tran, Thuy Khuong Le, Boi Ngoc Nguyen, Thao Nguyen, Nguyen Minh Nui, Tran Quang Nam, Kim Chi Tran

Supplemental Table 1. FIDELIO-DKD and FIGARO-DKD study details as published in the FIDELITY analysis^1^

| **Study name** | FIDELIO-DKD^2^ | FIGARO-DKD^3^ |
| --- | --- | --- |
| **Publication year** | 2020 | 2021 |
| **Study design** | Phase III, randomized, double-blind, placebo-controlled, multicenter clinical trial | Phase III, randomized, double-blind, placebo-controlled, multicenter clinical trial |
| **Sample size^a^** | 5,734 | 7,437 |
| **Inclusion criteria** | - Age ≥18 years - T2D and CKD defined as UACR 30-<300 mg/g, 25-<60 ml/min/1.73 m^2^, and diabetic retinopathy, *or* UACR 300-5000 and eGFR 25-<75 ml/min/1.73 m^2^ - Maximum tolerated dose of a RAS inhibitor - Serum potassium ≤4.8 mmol/l | - Age ≥18 years - T2D and CKD defined as UACR 30-<300 mg/g and eGFR 25-90 ml/min/1.73 m^2^, *or* UACR 300-5000 mg/g and eGFR ≥60 ml/min/1.73 m^2^ - Maximum tolerated dose of a RAS inhibitor - Serum potassium ≤4.8 mmol/l |
| **Exclusion criteria** | - Non-diabetic kidney disease - Uncontrolled hypertension^b^ - HbA1c >12% - SBP <90 mmHg - Chronic symptomatic HFrEF^c^ - Recent CV event - Dialysis for acute kidney failure - Kidney transplant | - Non-diabetic kidney disease - Uncontrolled hypertension^b^ - HbA1c >12% - SBP <90 mmHg - Chronic symptomatic HFrEF^c^ - Recent CV event - Dialysis for acute kidney failure - Kidney transplant |
| **Follow-up period, median** | 2.6 years | 3.4 years |
| **Primary outcome** | Time to kidney failure, a sustained decrease of at least 40% in eGFR from baseline, or renal death | Time to CV death, non-fatal MI, or stroke, or HHF |
| **Secondary outcome** | Time to CV death, non-fatal MI, or stroke, or HHF | Time to kidney failure, a sustained decrease of at least 40% in eGFR from baseline, or renal death |
| **Trial registry information** | [NCT02540993](https://clinicaltrials.gov/ct2/show/NCT02540993) | [NCT02545049](https://clinicaltrials.gov/ct2/show/NCT02545049) |

^a^A total of 145 randomized patients (60 patients in FIDELIO-DKD and 85 patients in FIGARO-DKD) were prospectively excluded prior to database lock from all analyses because of critical Good Clinical Practice violations. This affected one site in the USA that was subsequently closed during the conduct of the trial, leading to the exclusion of 66 patients. In addition, during trial conduct, it was detected that several patients were randomized simultaneously at multiple trial sites in the same locality in Florida, USA. This led to the prospective exclusion of a total of 79 patient IDs.

^b^Mean sitting SBP ≥70 mmHg or mean sitting DBP ≥110 mmHg at the run-in visit, or mean sitting SBP ≥160 mmHg or mean sitting DBP ≥100 mmHg at the screening visit.

^c^New York Heart Association class II-IV at the run-in visit.

CKD, chronic kidney disease; CV, cardiovascular; DBP, diastolic blood pressure; eGFR, estimated glomerular filtration rate; HbA1c, glycated hemoglobin; HFrEF; heart failure with reduced ejection fraction; HHF, hospitalization for heart failure; MI, myocardial infarction; RAS, renin–angiotensin system; SBP, systolic blood pressure; T2D, type 2 diabetes; UACR, urine albumin-to-creatinine ratio.

Supplemental Table 2. Baseline demographic and clinical characteristics of Hispanic and non-Hispanic patients by treatment allocation

| **Characteristic** | **Hispanic** | | **Non-Hispanic** | |
| --- | --- | --- | --- | --- |
|  | **Finerenone**  **(*N* = 1,065)** | **Placebo**  **(*N* = 1,034)** | **Finerenone**  **(*N* = 5,454)** | **Placebo**  **(*N* = 5,473)** |
| Age, years, mean ± SD | 64.2 ± 9.9 | 64.3 ± 9.6 | 64.8 ± 9.3 | 64.9 ± 9.7 |
| Sex, female, *n* (%) | 433 (40.7) | 374 (36.2) | 1,605 (29.4) | 1,526 (27.9) |
| Systolic blood pressure, mm Hg, mean ± SD | 137.0 ± 14.8 | 137.0 ± 14.7 | 136.8 ± 14.0 | 136.6 ± 14.2 |
| Diastolic blood pressure, mm Hg, mean ± SD | 76.7 ± 9.5 | 77.2 ± 9.4 | 76.3 ± 9.6 | 76.2 ± 9.6 |
| BMI, kg/m^2^, mean ± SD | 31.3 ± 6.3 | 31.3 ± 5.9 | 31.3 ± 6.0 | 31.3 ± 6.0 |
| Waist-hip ratio, mean ± SD | 1.0 ± 0.1 | 1.0 ± 0.2 | 1.0 ± 0.1 | 1.0 ± 0.1 |
| Waist circumference, cm, mean ± SD | 105.7 ± 14.7 | 105.9 ± 14.1 | 107.2 ± 15.2 | 107.3 ± 15.2 |
| Duration of diabetes, years, mean ± SD | 16.7 ± 9.2 | 16.4 ± 8.9 | 15.2 ± 8.6 | 15.2 ± 8.6 |
| HbA1c, %, mean ± SD | 8.0 ± 1.5 | 7.9 ± 1.5 | 7.7 ± 1.3 | 7.6 ± 1.3 |
| Serum potassium, mmol/l, mean ± SD | 4.40 ± 0.42 | 4.40 ± 0.4 | 4.34 ± 0.44 | 4.34 ± 0.44 |
| eGFR, ml/min/1.73 m^2^, mean ± SD | 60.6 ± 23.2 | 61.1 ± 22.4 | 56.9 ± 21.2 | 57.0 ± 21.6 |
| **eGFR, ml/min/1.73 m^2^, *n*/*N* (%)** | | | | |
| <45 | 330 (31.0) | 280 (27.1) | 1,868 (34.3) | 1,916 (35.0) |
| 45-<60 | 314 (29.5) | 266 (25.7) | 1,478 (27.1) | 1,452 (26.5) |
| ≥60 | 495 (46.5) | 488 (47.2) | 2,108 (38.7) | 2,104 (38.4) |
| UACR, mg/g, median, IQR | 569  (254-1,190) | 526  (241-1,189) | 501  (188-1,110) | 512  (190-1,157) |
| **UACR, mg/g, *n* (%)^i^** | | | | |
| <30 | 18 (1.7) | 23 (2.2) | 102 (1.9) | 87 (1.6) |
| 30-<300 | 287 (26.9) | 9,289 (27.4) | 1,789 (32.8) | 1,734 (31.7) |
| ≥300 | 759 (71.3) | 721 (69.7) | 3,562 (65.3) | 3,650 (66.7) |
| Current smoker, *n* (%) | 105 (9.9) | 101 (9.8) | 960 (17.6) | 927 (16.9) |
| History of CVD, *n* (%) | 639 (60.0) | 586 (56.7) | 2,901 (53.2) | 2,965 (54.2) |
| **Medication use at baseline, *n* (%)** | | | | |
| Angiotensin-converting enzyme inhibitors | 388 (36.4) | 351 (33.9) | 2,138 (39.2) | 2,202 (40.2) |
| Angiotensin receptor blockers | 677 (63.6) | 681 (65.9) | 3,310 (60.7) | 3,269 (59.7) |
| Beta-blockers | 466 (43.8) | 464 (44.9) | 2,770 (50.8) | 2,804 (51.2) |
| Diuretics | 500 (46.9) | 499 (48.3) | 2,825 (51.8) | 2,886 (52.7) |
| Statins | 742 (69.7) | 747 (72.2) | 3,915 (71.8) | 3,995 (73.0) |
| Potassium supplements | 11 (1.0) | 12 (1.2) | 185 (3.4%) | 177 (3.2) |
| Potassium-lowering agents (including binders) | 2 (0.2) | 3 (0.3) | 92 (1.7) | 85 (1.6%) |
| Glucose-lowering therapies | 1,048 (98.4) | 1,011 (97.8) | 5,306 (97.3) | 5,306 (97.3) |
| Insulin and analogues | 674 (63.3) | 674 (63.3) | 3,192 (58.5) | 3,192 (58.5) |
| Metformin | 686 (64.4) | 686 (64.4) | 3,126 (57.3) | 3,126 (57.3) |
| Sulfonylureas | 258 (24.2) | 258 (24.2) | 1,433 (26.3) | 1,433 (26.3) |
| DPP-4 inhibitors | 184 (17.3) | 184 (17.3) | 1,476 (27.1) | 1,476 (27.1) |
| GLP-1RAs | 36 (3.4) | 36 (3.4) | 461 (8.5) | 461 (8.5) |
| SGLT-2is | 59 (5.5) | 59 (5.5) | 379 (6.9) | 379 (6.9) |

BMI, body mass index; CVD, cardiovascular disease; DPP-4, dipeptidyl peptidase-4; eGFR, estimated glomerular filtration rate; GLP-1RA, glucagon like peptide-1 receptor agonist; HbA1c, glycated hemoglobin; IQR, interquartile range; SD, standard deviation; SGLT-2i, sodium-glucose co-transporter-2 inhibitor; UACR, urine albumin-to-creatinine ratio

Supplemental Table 3. Medical history findings of interest at baseline by treatment allocation

| **Patients with medical history finding of interests, *n* (%)** | **Hispanic** | | **Non-Hispanic** | |
| --- | --- | --- | --- | --- |
|  | **Finerenone**  **(*N* = 1,065)** | **Placebo**  **(*N* = 1,034)** | **Finerenone**  **(*N* = 5,454)** | **Placebo**  **(*N* = 5,473)** |
| **Cardiovascular** | | | | |
| Hypertension | 1,016 (95.4) | 989 (95.6) | 5,265 (96.5) | 5,296 (96.8) |
| Hyperlipidemia | 322 (30.2) | 319 (30.9) | 2,474 (45.4) | 2,511 (45.9) |
| Coronary artery disease | 242 (22.7) | 252 (24.4) | 1,748 (32.0) | 1,755 (32.1) |
| Peripheral arterial occlusive disease | 151 (14.2) | 168 (16.2) | 906 (16.6) | 860 (15.7) |
| Myocardial infarction | 179 (16.8) | 177 (17.1) | 839 (15.4) | 827 (15.1) |
| Ischemic stroke | 90 (8.5) | 102 (9.9) | 681 (12.5) | 683 (12.5) |
| Atrial fibrillation and atrial flutter | 40 (3.8) | 35 (3.4) | 528 (9.7) | 503 (9.2) |
| Cardiac failure | 44 (4.1) | 64 (6.2) | 441 (8.1) | 458 (8.4) |
| Percutaneous coronary intervention | 41 (3.8) | 29 (2.8) | 327 (6.0) | 304 (5.6) |
| Coronary artery bypass graft | 26 (2.4) | 37 (3.6) | 322 (5.9) | 311 (5.7) |
| Periodontal disease | 30 (2.8) | 22 (2.1) | 269 (4.9) | 280 (5.1) |
| Carotid endarterectomy | 6 (0.6) | 12 (1.2) | 70 (1.3) | 76 (1.4) |
| **Diabetic** | | | | |
| Diabetic retinopathy | 344 (32.3) | 330 (31.9) | 2,161 (39.6) | 2,119 (38.7) |
| Diabetic neuropathy | 242 (22.7) | 219 (21.2) | 1,546 (28.3) | 1,493 (27.3) |


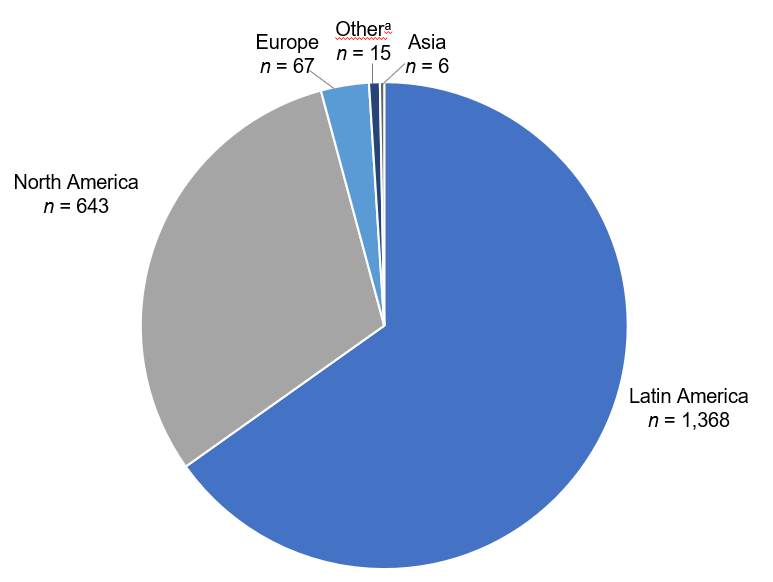
Supplemental Figure 1. Distribution of randomized subjects in the Hispanic subgroup. ^a^Other includes Australia, New Zealand, and South Africa.


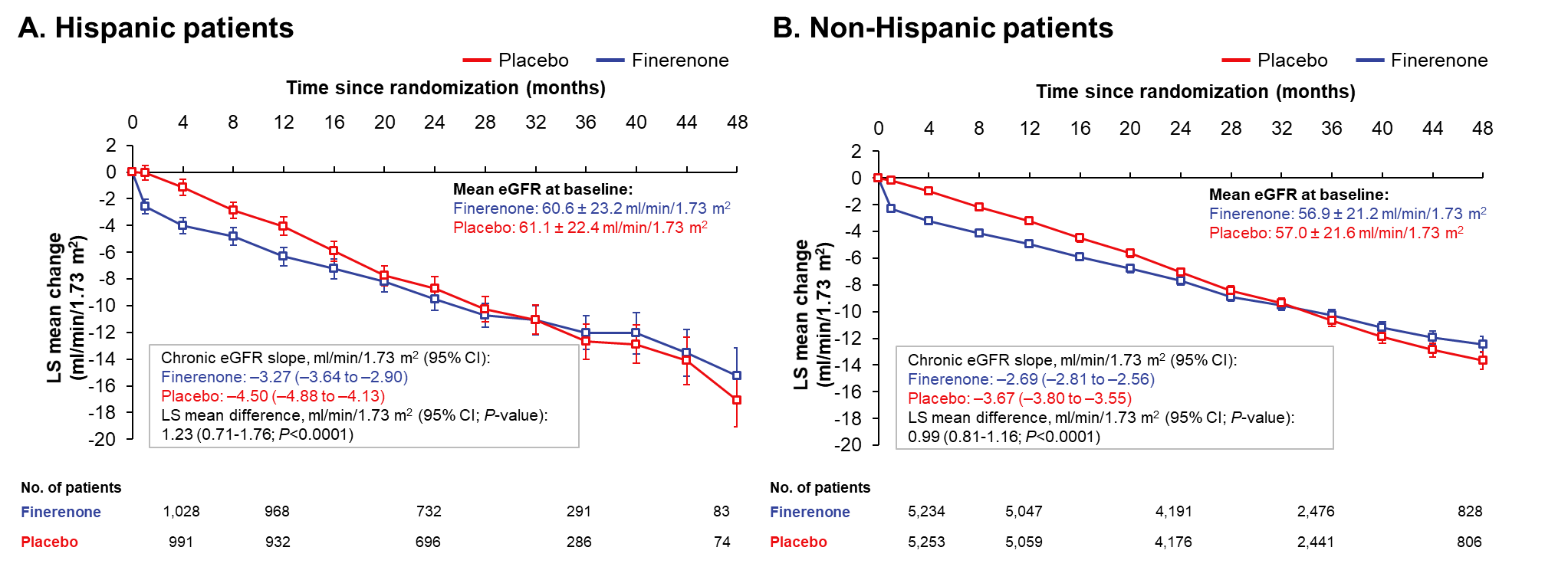


Supplemental Figure 2. Change in eGFR over time in Hispanic and non-Hispanic patients. CI, confidence interval; eGFR, estimated glomerular filtration rate; LS, least-squares.


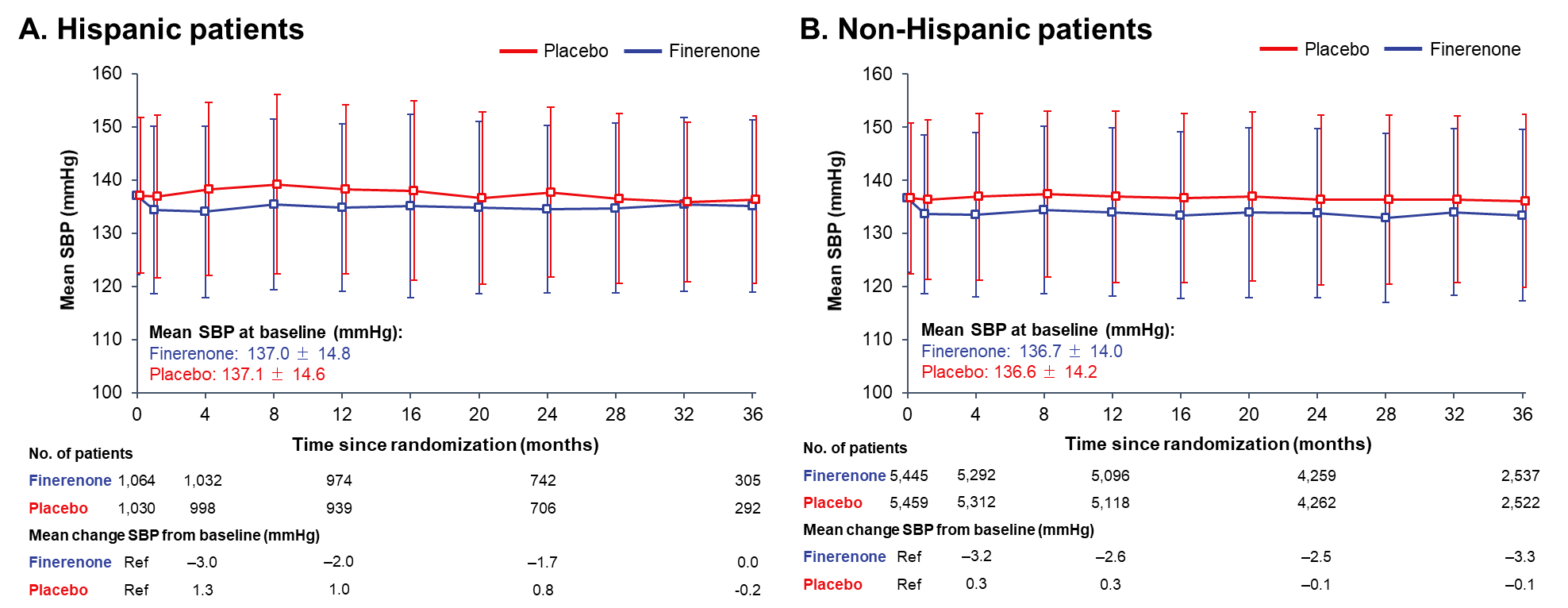


Supplemental Figure 3. Mean change in SBP over time in Hispanic and non-Hispanic patients. SBP, systolic blood pressure.

References

1. Agarwal R, Filippatos G, Pitt B, et al. Cardiovascular and kidney outcomes with finerenone in patients with type 2 diabetes and chronic kidney disease: the FIDELITY pooled analysis. European heart journal 2022;43(6):474–484.

2. Bakris GL, Agarwal R, Anker SD, et al. Effect of finerenone on chronic kidney disease outcomes in type 2 diabetes. N Engl J Med 2020;383:2219–2229.

3. Pitt B, Filippatos G, Agarwal R, et al. Cardiovascular events with finerenone in kidney disease and type 2 diabetes. N Engl J Med 2021;385(24):2252–2263.
